# The pharmacokinetics, bioavailability and excretion of bergapten after oral and intravenous administration in rats using high performance liquid chromatography with fluorescence detection

**DOI:** 10.1186/s13065-016-0212-x

**Published:** 2016-10-14

**Authors:** Xie-an Yu, John Teye Azietaku, Jin Li, Mingrui An, Jun He, Jia Hao, Jun Cao, Yan-xu Chang

**Affiliations:** 1 Tianjin State Key Laboratory of Modern Chinese Medicine, Tianjin University of Traditional Chinese Medicine, Tianjin, 300193 China; 2 Department of Surgery, University of Michigan, Ann Arbor, MI 48109 USA; 3 College of Material Chemistry and Chemical Engineering, Hangzhou Normal University, Hangzhou, 310036 China

**Keywords:** HPLC-FLD, Bergapten, Oral bioavailability and excretion

## Abstract

A sensitive, specific, reproducible and optimized high performance liquid chromatography with fluorescence detection (HPLC-FLD) method for the determination of bergapten in rat plasma was established and applied to the pharmacokinetic and bioavailability study in rat after oral and intravenous administration of bergapten. The method was also successfully applied to the excretion study of bergapten after an oral administration of bergapten at a dose of 15 mg kg^−1^ to rats. The sample preparation was achieved using liquid–liquid extraction. Isoimperatorin was used as the internal standard (IS). The analytes were detected by using fluorescence detection at an excitation and emission wavelength of 288 and 478 nm, respectively. Using aqueous formic acid (0.1 %, v/v) and acetonitrile as the mobile phase, the chromatographic separation was achieved on a Hedera™ ODS column at a flow rate of 1 mL min^−1^. The lower limit of quantitation (LLOQ) of bergapten was 2 ng mL^−1^. The HPLC-FLD method was successfully applied to the pharmacokinetic, bioavailability and excretion study of bergapten in rats.

**Graphical abstract:**
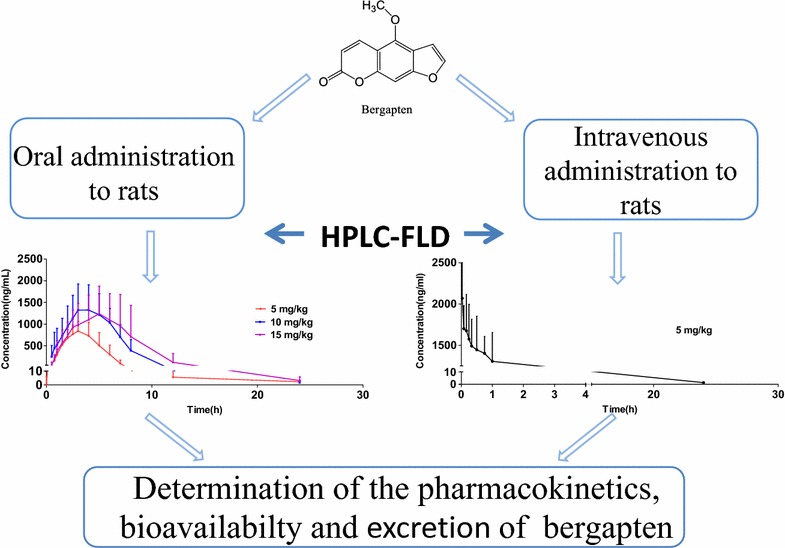
An high performance liquid chromatography with fluorescence detection (HPLC-FLD) method for the pharmacokinetic and bioavailability study in rat after administration of bergapten.

An high performance liquid chromatography with fluorescence detection (HPLC-FLD) method for the pharmacokinetic and bioavailability study in rat after administration of bergapten.

## Background

**Fig. 1:**
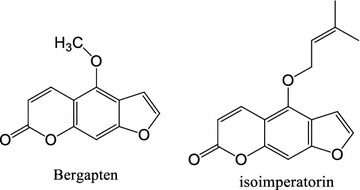
Chemical structures of bergapten and isoimperatorin (IS)

Bergapten (Fig. 1), is one of coumarins found in many herbal medicines. Pharmacological studies showed that bergapten had the analgesic, anti-inflammatory, anti-coagulant and anti-cancer activities [1, 2]. Bergapten has also been known to counteract the proliferative effect and cause apoptosis of breast cancer cells [3]. Previous studies have shown that bergapten reduced the level of circulating estrogen and improved oxidative metabolism [4]. Several analytical methods for investigating coumarins in biological fluids have been previously reported [5, 6]. Many of these methods on bergapten focused on the simultaneous determination of two or more compounds including bergapten using HPLC–UV [7], LC–MS [4, 5] and high-speed countercurrent chromatography [8]. Currently, an LC–MS/MS method was developed to determine bergapten in dog plasma [9]. To the best of our knowledge, no article has focused on oral bioavailability and excretion study of pure compound of bergapten in rats.

Fluorometric analysis is among the most sensitive and selective methods for detecting organic and inorganic compounds. Coumarins have been known to be interesting fluorophores, with their fluorescences changing drastically with substituents and their introduced positions [10]. In this present study, a simple, selective, sensitive and optimised HPLC-FLD method has been developed for the quantitative determination of bergapten in rat plasma using isoimperatorin as an internal standard (IS). This analytical method has been successfully applied to the pharmacokinetics, oral bioavailability and excretion studies of bergapten after oral and intravenous administration to rats. This is an oral bioavailability and excretion study that have been reported on bergapten in rats after a search into various journals.

## Methods

### Chemicals and reagents

Acetonitrile (Fisher technologies Inc., USA) and methanol (Tianjin concord Science Co. Ltd., Tianjin, China) were of HPLC grade. Standard reference isoimperatorin and bergapten (purity >98 %) were purchased from National Institute for the Control of Pharmaceutical and Biological Products (Beijing, China). Ethyl acetate and formic acid were of analytical grade. Deionized water was purified with a Milli-Q Academic ultra-pure water system (Millipore, Milford, MA, USA) and used for the HPLC mobile phase.

### Apparatus and chromatographic conditions

HPLC analysis was performed on an Agilent 1100 HPLC (Agilent Technologies, USA) equipped with a quaternary pump, a degasser, an autosampler, a column thermostat and a fluorescence detector. An agilent fluorescence detector was coupled to the Agilent system. Separation was carried out with a Hedera™ ODS column (4.6 × 250 mm, 5 μm) by gradient elution at a temperature of 30 °C. Excitation and emission of the fluorescence detector was set to 288 and 478 nm, respectively. A constant flow rate of 1.0 mL min^−1^ and an injection volume of 30 μL were employed throughout the analysis. A mobile phase comprising of aqueous formic acid (0.1 %, v/v) (solvent system A) and acetonitrile (solvent system B) was employed with a gradient elution of 40–80 % B at 0 to 5 min, 80–85 % B at 5 to 10 min, 85–90 % B at 10 to 12 min, 90–95 % B at 12–15 min, 95 % B at 15–20 min. The re-equilibration time of gradient elution was 8 min.

### Preparation of stock solution, calibration standards

In preparing the stock solution, appropriate amount of bergapten was weighed and dissolved in methanol to achieve a concentration of 1.0 mg mL^−1^. The chemical structures of bergapten and isoimperatorin are shown in Fig. 1. Working solutions of bergapten were then prepared by appropriate dilution with methanol for use. The stock solution of internal standard, isoimperatorin was also dissolved in methanol and diluted with methanol to a final concentration of 1 μg mL^−1^ and stored at 4 °C until analysis.

10 μL aliquots of bergapten working solutions were added to 100 μL drug-free rat plasma to obtain bergapten calibration standards (2, 4, 8, 20, 40, 100 and 100, 200, 500, 1000, 2500, 5000 ng mL^−1^) in plasma samples for two calibration curves.

### Sample pretreatment and quality samples

To a 100 μL aliquot of plasma sample, 10 μL internal standard solutions were added. Samples were vortex-mixed for 2 min, extracted with 1000 μL ethyl acetate and then centrifuged for 10 min at 14,000 rpm. The supernatant was transferred into another centrifuge tube and evaporated to dryness using nitrogen gas. The dried residue was reconstituted by adding 100 μL methanol. The solution was shaken and ultrasonicated for 2 min. It was then centrifuged at 14,000 rpm for 10 min. A 30 μL of the solution was run with the HPLC and analysis was performed.

For the quality control (QC) samples (2, 6, 500 and 5000 ng mL^−1^), blank rat plasma was spiked with appropriate standard solutions of bergapten to the required plasma concentrations, followed by the same sample preparation and extraction method described above.

### Method validation

Testing for specificity involved comparing the chromatograms of six different batches of blank rat plasma samples with that of their corresponding spiked plasma. The limit of detection (LOD) was defined as the amount of analyte that could be detected with a signal to noise ratio of 3. The lower limit of quantification (LLOQ), which is the lowest concentration in the standard curve at which the signal to noise ratio (S/N) was to be larger than 5, with relative standard deviation (RSD n = 6) within 20 % and accuracy in the range of 80 % to 120 according to the guidelines for industry (2001). In determining the linearity of the method, samples were prepared by spiking blank rat plasma with standard solutions (prepared in methanol) of bergapten to the concentrations: 2, 4, 8, 20, 40, 100 and 100, 200, 500, 1000, 2500 and 5000 ng mL^−1^ for the calibration curves. In determining the intra-day accuracy and precision, four quality control (QC) samples (n = 6) were assayed within the same day. This was in turn repeated once a day for 3 consecutive days to evaluate the inter-day precision along with the standard calibration curve. The determination of the extraction recoveries was performed by comparing the observed peak areas of bergapten in extracted plasma samples with those of the bergapten in non-processed plasma samples at the same theoretical concentrations. The tests for stability were investigated for bergapten in autosampler for 24 h, after 3 times freeze and thaw cycles and also after storing in a freezer at a temperature of −20 °C for 1 month.

### Application to a pharmacokinetic study in rats

Male Sprague–Dawley rats (240–260 g) were fed with standard laboratory food and water and kept in an environmentally-controlled breeding room for at least 1 week before experimentation. The rats were fasted for 12 h and allowed free access to water prior to the experiments. The rats were randomly divided into 4 groups with eight rats in each group to diminish the individual variation. The first group was given bergapten intravenously at a dose of 5 mg kg^−1^ while the other three groups were given bergapten orally at doses of 5, 10 and 15 mg kg^−1^. Disposable sterilized syringes were used for intravenous administration and medical cotton ball was pressured on the wound until bloodless after injection. Blood samples (about 250 μL) were immediately collected in heparinized 1.5 mL polythene tubes from the suborbital vein at 0, 0.5, 0.75, 1, 1.5, 2, 2.5, 3, 4, 5, 6, 7, 8, 12, 24 h after oral administration. For intravenous administration, time intervals were set at 0, 0.033, 0.083, 0.17, 0.25, 0.33, 0.5, 0.75, 1, 2, 3, 4, 6, 8, 12, 24 h for blood sampling. All blood samples were immediately centrifuged to separate plasma at 6000 rpm for 10 min. The plasma was transferred into clean tubes and stored at −20 °C until analysis. Animal welfare and experimental procedures were strictly in accordance with the guide for the care and use of laboratory animals and the related ethical regulations of Tianjin University of Traditional Chinese Medicine.

### Excretion of bergapten in rat urine, feces and bile

Sixteen male Sprague–Dawley (SD) rats (250 ± 10 g) were divided into two groups (group1 were for collecting urine and fecal samples in metabolic cages while group 2 were for collecting bile samples from the bile duct using polyethylene tubes). For group 1, the rats were orally administrated with bergapten dissolved in 0.5 % CMC-Na at a dosage of 15 mg kg^−1^ and placed in metabolic cages enabling collection of urine and fecal samples separately. The urine and fecal samples were collected at time interval of 0–4, 4–8, 8–12, 12–24, 24–36, 36–48, 48–60, and 60–72 h. For Group 2, the rats were anaesthetised with chloral hydrate at a dose of 0.3 g kg^−1^ administered intraperitoneally. A polyethene tube was used in cannula ting the bile duct ensuring continuous flow of bile. Bile samples were then collected at different time intervals (0–1, 1–2, 2–3, 3–4, 4–5, 5–6, 6–7, 7–8, 8–9 and 9–10 h). After the volumes of urine and bile obtained were measured, these samples were stored at −20 °C until analysis. The preparation of the urine and bile samples were the same as the plasma sample preparation described above. The fecal samples, on the other hand, after collection were dried out in a drying oven at 40 °C. After measuring the weights of the fecal samples, they were crushed by a mortar to achieve a uniform powder. 0.1 g of powdered feces was measured and 1 mL methanol was added in 1.5 mL polythene tubes, mixed sufficiently for 3 min by vortexing and extracted ultrasonically for 30 min. Supernatant were transferred into vials for analysis using HPLC-FLD.

### Data analysis

The DAS software (Drug and Statistics 1.0, Medical College of Wannan, China), a computer program was used in calculating the pharmacokinetic parameters after administering bergapten both intravenously and orally at dose of 5 and 5, 10, 15 mg kg^−1^, respectively. To choose the optimum compartment model for fitting the plasma concentration–time curve, the minimum Akaike’s information criterion (AIC) estimation was tested by calculating the lowest AIC value. The compartment model with minimum AIC is regarded as the best representation of the plasma concentration–time course data [10]. The bioavailability was calculated as follows: F = (AUC_oral_/AUC_intravenous_) × 100 %. Both AUC oral and AUC intravenous were estimated by one-compartment model.

## Results and discussion

### Optimization of the fluorescence spectra

The excitation and emission wavelengths of bergapten were optimized to obtain a suitable detection wavelength with an increased signal to noise (S/N). After several examinations, an excitation wavelength of 288 nm and emission wavelength of 478 nm was the most suitable fluorescence detection wavelength for bergapten and the IS isoimperatorin.

### Method validation

#### Specificity

**Fig. 2:**
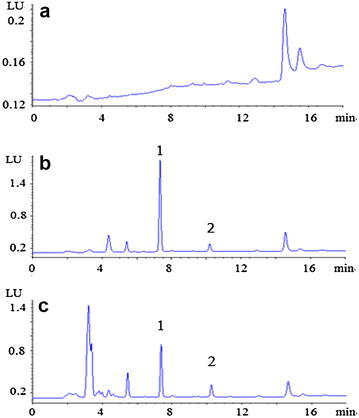
Representative chromatogram of **a** blank rat plasma, **b** blank rat plasma spiked with standard compounds, and **c** real sample after oral administration 5 mg kg^−1^of bergapten. *1* bergapten, *2* isoimperatorin

Figure 2 shows the representative chromatograms of blank plasma, blank plasma samples spiked with bergapten and plasma sample obtained from a rat following an injection of bergapten. The retention time of bergapten was 7.3 min and IS was 10.2 min. As described above, good resolution was achieved between analyte and IS and no substance from several different sources of rat plasma was observed interfering with the separation and quantitation of bergapten. In the real pharmacokinetic study samples, no metabolite or endogenous substance interfered with the determination of the analytes.

### Calibration curve and lower limits of quantification

The model of calibration for the two calibration curves was selected based on the analysis of the data by linear regression and with weighting factor (1/*x*). The peak area ratio of bergapten to IS in rat plasma was linear in relation to the concentration of the analyte for the ranges, 2–100 ng mL^−1^ and 100–5000 ng mL^−1^. The regression equation for calibration one was Y = 0.006581X − 0.00793 (correlation coefficient, r = 0.9990), and that for the second calibration was Y = 0.007403X + 0.050226 (correlation coefficient, r = 0.9992) over the range 100-5000 ng mL^−1^. The LOD for bergapten was found to be 1 ng mL^−1^ (S/N ≥ 3) and LLOQ was 2 ng mL^−1^(S/N ≥ 5).

#### Accuracy and precision

Both intra-day and inter-day accuracy and precision values of the method are shown in Table 1. The intra-day coefficient of variation (RSD %) for bergapten ranged from 4.29 to 11.6 % and the accuracy from 97.4 to 109 %. RSD of the inter-day for the analyte was from 7.13 to 13.6 % and the accuracy from 93.7 to 107 %. The results indicated that the assay was reproducible, accurate and reliable.

**Table 1:** Intra-day, inter-day accuracy and precision of bergapten (n = 6)

Concentration (ng mL^−1^)	Intra-day		Inter-day	
	Accuracy (%)	Precision (%)	Accuracy (%)	Precision (%)
2	97.4	8.92	93.7	7.13
6	103	11.6	99.4	13.1
500	108	6.34	107	9.97
5000	109	4.29	106	13.6

#### Extraction efficiency

The recovery of bergapten was higher than 80 % at all the four concentrations studied (Table 2) and the extraction efficiency did not show obvious dependent relation with concentration. It was concluded that liquid–liquid extraction with ethyl acetate proved to be efficient in extracting bergapten from the plasma sample.

**Table 2:** Recoveries of bergapten (n = 6)

Concentration (ng mL^−1^)	Recovery	
	Accuracy (%)	RSD (%)
2	84.2	6.42
6	101	10.0
500	110	7.49
5000	112	6.82

#### Stability

The results from the stability tests are shown in Table 3. It was found that bergapten was stable in rat plasma after three freeze–thaw cycles. Bergapten was also stable in the auto-sampler for a period of 24 h. The reduction of bergapten content in the rat plasma observed under any of those conditions was not significant. The above results demonstrated that bergapten could be determined in rat plasma by the developed HPLC method.

**Table 3:** Stability of bergapten (n = 6)

Concentration (ng mL^−1^)	Freeze thaw cycles		Autosampler for 24 h		−20 °C for 1 month	
	Accuracy (%)	RSD (%)	Accuracy (%)	RSD (%)	Accuracy (%)	RSD (%)
2	89.5	11.9	107	8.69	97.6	7.33
6	101	13.1	110	7.49	96.9	7.71
500	113	5.65	102	4.31	90.5	5.99
5000	92	10.9	91.7	4.85	99.4	9.31

### Pharmacokinetic studies

#### Pharmacokinetics of bergapten in rats after intravenous administration

**Fig. 3:**
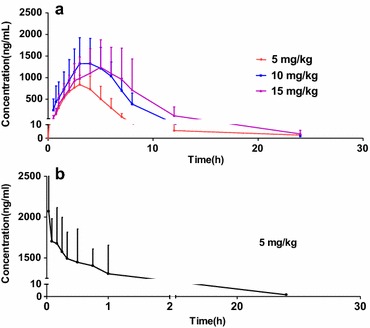
The mean plasma concentration–time profiles of bergapten after oral (**a**) and intravenous (**b**) administration (n = 8, mean ± SD)

The pharmacokinetics of bergapten in rat plasma after administering bergapten both intravenously and orally was successfully studied using the developed method. After intravenous administration of bergapten at dose of 5 mg kg^−1^ to rats, the mean plasma concentration–time profile of bergapten is shown in Fig. 3. Some important pharmacokinetic parameters have been listed in Table 4. The distribution of bergapten into the tissues was slow and this is indicated by the long distribution half-life, T_1/2α_ of 2 h. The plasma concentration of bergapten decreased gradually within the first 4 h after interavenous administration, and then slowly decreased to the LLOQ during the next 8 h. The average volume of distribution is 0.0027 ± 0.0006 L Kg^−1^. The mean area for the plasma concentration–time curve from time zero to the last measurable plasma concentration point (AUC_0–tn_) was 4391 ± 1363 ng (mL h)^−1^ and the mean area under the plasma concentration–time curve from time zero to time infinity (AUC_0–∞_) was 4474 ± 1322 ng (mL h)^−1^.

**Table 4:** Pharmacokinetic parameters of bergapten after intravenous administration of 5 mg kg^−1^ (n = 8, mean ± SD)

Parameters	Low (5 mg kg^−1^)
T_max_ (h)	0.0333
C_max_ (ng mL^−1^)	2080 ± 484
AUC_(0–tn)_ (ng mL^−1^ h^−1^)	4391 ± 1363
AUC_(0–∞)_ (ng mL^−1^ h^−1^)	4474 ± 1323
V/F (L)	0.0027 ± 0.0006
T1/2α (h)	1.74 ± 0.21
MRT_(0–tn)_ (h)	1.80 ± 0.10
MRT_(0–∞)_ (h)	4.05 ± 3.81

#### Pharmacokinetics of bergapten in rats after oral administration

After administering bergapten orally at doses of 5, 10 and 15 mg kg^−1^, the mean plasma concentration–time curves are illustrated in Fig. 3. The major pharmacokinetic parameters of bergapten are presented in Table 5. After oral administration, the absorption of bergapten from the rat gastrointestinal tract was discovered to be rapid. It was detected in the plasma after the first blood was sampled at a time of 2 min and T_max_ was reached slightly rapid for all three oral doses studied. The plasma concentration then decreased to the LLOQ by 24 h. It was also observed that the C_max_ and AUC after administering bergapten at doses 10 and 15 mg kg^−1^ were similar. It could be inferred here that this concentration was the allowable amount of bergapten that can be absorbed by the rats. According to the pharmacokinetic calculations by DAS 1.0 software, which is the authoritative software for the pharmacokinetic calculations, a one-compartment model of in vivo metabolism best fit the data on bergapten in rats after oral administration.

**Table 5:** Pharmacokinetic parameters of bergapten after oral administration of 5, 10, 15 mg kg^−1^ (n = 8, mean ± SD)

Parameters	Low (5 mg kg^−1^)	Medium (10 mg kg^−1^)	High (15 mg kg^−1^)
T_max_ (h)	3.20 ± 0.45	3.88 ± 0.99	4.56 ± 1.40
C_max_ (ng mL^−1^)	859.4 ± 253.6	1397 ± 573	1307 ± 617
AUC _(0–tn)_ (ng mL^−1^ h^−1^)	3517 ± 1299	8255 ± 3536	9197 ± 5790
AUC_(0–∞)_ (ng mL^−1^ h^−1^)	3537 ± 1302	8266 ± 3534	9306 ± 5782
V/F (L)	0.0107 ± 0.0044	0.0115 ± 0.0139	0.0124 ± 0.0138
T_1/2α_ (h)	9.35 ± 3.07	12.88 ± 12.21	14.35 ± 15.75
MRT_(0–tn)_ (h)	3.72 ± 0.53	4.83 ± 0.47	5.57 ± 1.15
MRT_(0–∞)_ (h)	3.91 ± 0.51	4.87 ± 0.47	6.65 ± 2.27

The mean area under curve AUC_(0–tn)_ from time 0 to 24 h were 3517 ± 1299, 8255 ± 3536, 9197 ± 5790 ng (L h)^−1^ and the mean area under the curve from time zero to time infinity AUC_(0–∞)_ were 3537 ± 1302, 8266 ± 3534, 9306 ± 5782 ng (L h)^−1^ for 5, 10 and 15 mg kg^−1^ doses, respectively. On the other hand, apparent volume of distribution (V) value was 0.02 L kg^−1^ for the oral group, suggesting that this compound could not distribute extensively into organs and tissues. The distribution half-life is 9 h and the MRT is 4 h.

### Bioavailability of bergaten in rats after administration

The absolute oral bioavailability (F) were 80.1 ± 29.6 %, 94.0 ± 40.3 % and 69.5 ± 44.2 % for low, medium and high concentrations using the formula F = (AUC_oral_/AUC_intravenous_) × 100 %, based on the AUC_(0–∞)_ obtained after intravenous and oral administration. The AUC of the medium and high concentration were similar, it could be inferred that the absorption of bergapten reached its peak within the range of 10 to 15 mg kg^−1^. It was demonstrated that bergapten might have a good absorption from the gastrointestinal tract in rat. It was also concluded that oral administration of bergapten may be the better route if it was developed the new drugs used in clinic.

### Excretion study of bergapten in rat urine, feces and bile

**Fig. 4:**
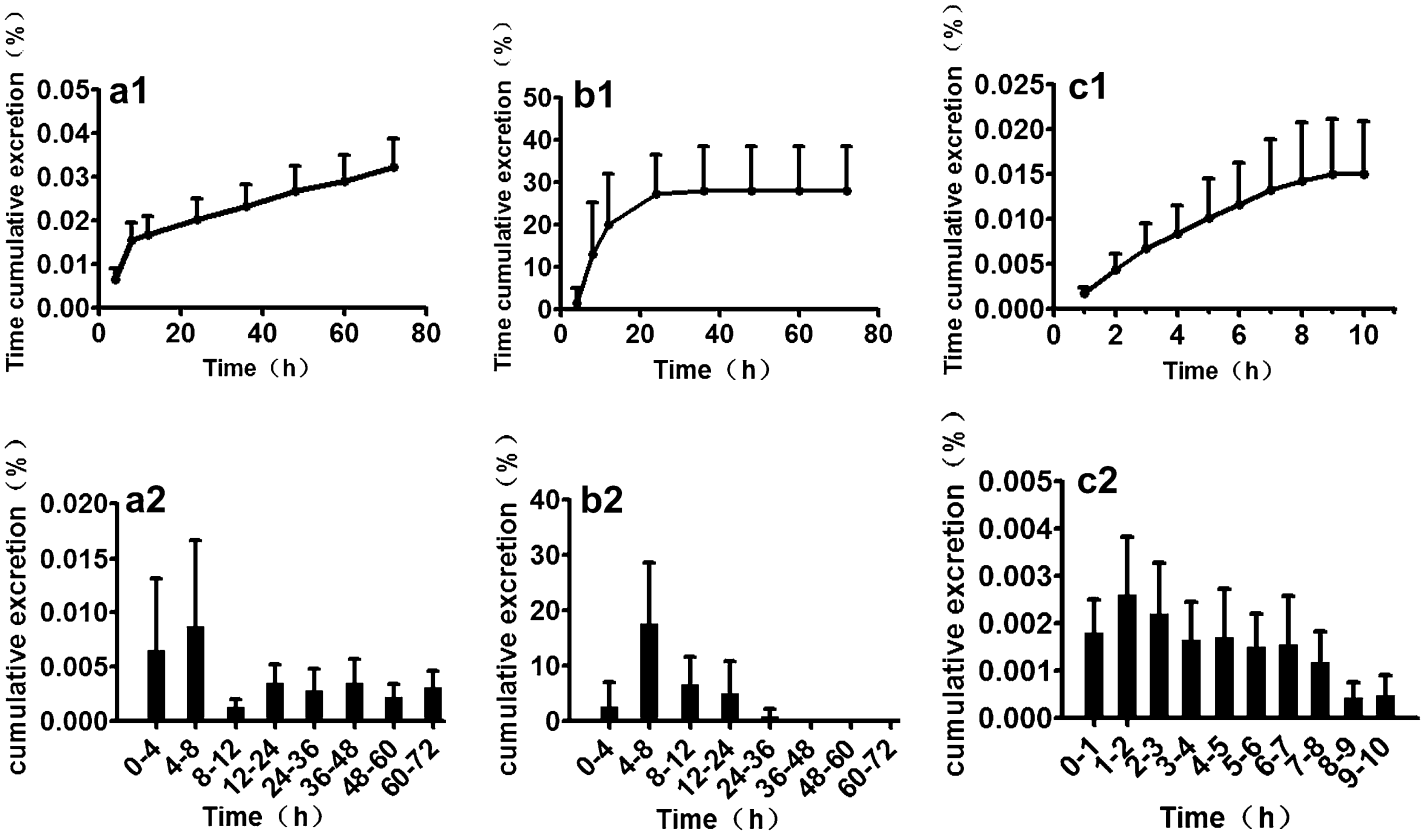
Time cumulative excretion percentage of bergapten (**a1**) in urine, (**b1**) in feces and (**c1**) in bile; cumulative excretion percentage at different time of bergapten (**a2**) in urine, (**b2**) in feces and (**c2**) in bile

The cumulative excretion of bergapten in urine, feces and bile were determined as shown in Fig. 4. After an oral administration of bergapten at a dose of 15 mg kg^−1^ to the rats, bergapten could be detected in rat urine until 72 h. Bergapten increased rapidly in urine during a time period of 4–8 h. After 8 h however, there was a gradual increase of bergapten in urine. Bergapten exhibited an increased rise in fecal sample up until a period of 36 h, after which the detection of bergapten was stable with minimal increase or decrease until 72 h. The rise was rapid especially in the period of 4–8 h. Therefore, the time cumulative excretion percentage of bergapten in feces stabilized after 36 h. Owing to the rats’ physical conditions, bile samples were only collected for a period of 10 h. After a more gradual increase in bile for a period of 8 h, bergapten stabilized. As shown in Fig. 4, the time cumulative amounts of bergapten in feces were 27.99 ± 10.08 % of the total dose, demonstrating that bergapten was mainly excreted in the feces. Even though the time cumulative amount of bergapten in urine was 0.032 ± 0.019 %, it continued to increase in urine until 72 h.

## Discussion

### Pharmacokinetic study of bergapten

The development of sensitive and specific assay of a drug is crucial to the study of drug pharmacokinetics. The HPLC-FLD was first developed to monitor the concentration of bergapten in solution to determine its suitability and sensitivity. The method was further optimized for the determination of bergapten in the rat plasma and has been validated to be sensitive to investigate the pharmacokinetics of bergapten in rats. Bergapten is an important furocoumarin because of its presence in many TCMs and the various therapeutic effects it possesses. Bergapten was rapidly absorbed by rats with the maximum plasma concentration achieved within 3 h after dosing (5 mg kg^−1^) as seen in Table 1. The kinetic properties were fit to the one-compartment model after rats were given i.v. administration. The absorption *T*_1/2_ after oral administration was 30 s, which shows that bergapten was also rapidly absorbed. The long distribution half-life could explain the reason for high bioavailability in rats after oral administration (T_1/2α_ (h) = 9.35 ± 3.06). The oral absolute bioavailability were 80.1 ± 29.6 %, 94.0 ± 40.3 % and 69.5 ± 44.2 % for low, medium and high concentration of bergapten, which showed that bergapten was provided with a higher degree of absorption from the gastrointestinal tract.

### Method comparison with existing reports

A pharmacokinetic study of bergapten was performed in dog plasma using an LC–MS/MS method. Tmax and AUC_(0–∞)_ were 4.2 h and 3219.2 ± 211.4 ng (mL h)^−1^ respectively which were comparable to that obtained from our experiment, giving a T_max_ and AUC_(0–∞)_ of 3–4.5 h and 3537 ± 1302 ng (mL h)^−1^. The LLOQ as obtained from the LC–MS/MS experiment was 0.5 ng mL^−1^ which differs from this study which was 2 ng mL^−1^ [8]. Our experiment differs from the LC–MS/MS experiments done on bergapten in that we determined the oral bioavailability and excretion of bergapten in rats. HPLC-FLD offers a cheaper analytical tool option compared to the higher cost of LC–MS/MS and HPLC-FLD requires less technical know-how.

## Conclusion

In this study, a sensitive, specific, reproducible and optimized HPLC-FLD method for the determination of bergapten in rat plasma was established and applied to the pharmacokinetic, bioavailability and excretion studies in rat after administering bergapten orally and intravenously to the rats. The method was thoroughly validated over two ranges of concentration of 2–100 ng mL^−1^ and 100–5000 ng mL^−1^ (r > 0.999) which produced a good intra-day and inter-day accuracy and precision. The sample preparation technique used was simple. The pharmacokinetics of bergapten follows a one compartment model and was well absorbed after oral administration. Base on the high bioavailability of bergapten after oral administration in rat, it was suggested that the better route of bergapten in clinic was oral administration. Cumulative excretion of bergapten in urine, feces and bile reached 0.032 ± 0.019 %, 27.99 ± 10.08 % and 0.015 ± 0.006 % of the total dosage, respectively. The excretion of bergapten was mainly through fecal route. For the first time, the oral bioavailability and excretion study of bergapten were reported in rats using HPLC-FLD method, which will provide more useful information on bergapten in in vivo pharmacological investigation and the new drug research. Pharmacokinetic and bioavailability study on bergapten can also be applied in evaluating the clinical efficiency of bergapten as used in clinic.
